# Characterization and tissue localization of zebrafish homologs of the human *ABCB1* multidrug transporter

**DOI:** 10.1038/s41598-021-03500-8

**Published:** 2021-12-17

**Authors:** Robert W. Robey, Andrea N. Robinson, Fatima Ali-Rahmani, Lyn M. Huff, Sabrina Lusvarghi, Shahrooz Vahedi, Jordan M. Hotz, Andrew C. Warner, Donna Butcher, Jennifer Matta, Elijah F. Edmondson, Tobie D. Lee, Jacob S. Roth, Olivia W. Lee, Min Shen, Kandice Tanner, Matthew D. Hall, Suresh V. Ambudkar, Michael M. Gottesman

**Affiliations:** 1 Laboratory of Cell Biology, Center for Cancer Research, National Cancer Institute, National Institutes of Health, Bethesda, MD USA; 2 Molecular Histopathology Laboratory, Frederick National Laboratory for Cancer Research, Frederick, MD USA; 3 National Center for Advancing Translational Sciences, National Institutes of Health, Rockville, MD USA

**Keywords:** Cell biology, Molecular biology

## Abstract

Capillary endothelial cells of the human blood–brain barrier (BBB) express high levels of P-glycoprotein (P-gp, encoded by *ABCB1*) and ABCG2 (encoded by *ABCG2*). However, little information is available regarding ATP-binding cassette transporters expressed at the zebrafish BBB, which has emerged as a potential model system. We report the characterization and tissue localization of two genes that are similar to *ABCB1,* zebrafish *abcb4* and *abcb5.* When stably expressed in HEK293 cells, both Abcb4 and Abcb5 conferred resistance to P-gp substrates; however, Abcb5 poorly transported doxorubicin and mitoxantrone compared to zebrafish Abcb4. Additionally, Abcb5 did not transport the fluorescent P-gp probes BODIPY-ethylenediamine or LDS 751, while they were transported by Abcb4. High-throughput screening of 90 human P-gp substrates confirmed that Abcb4 has an overlapping substrate specificity profile with P-gp. In the brain vasculature, RNAscope probes for *abcb4* colocalized with staining by the P-gp antibody C219, while *abcb5* was not detected. The *abcb4* probe also colocalized with claudin-5 in brain endothelial cells. Abcb4 and Abcb5 had different tissue localizations in multiple zebrafish tissues, potentially indicating different functions. The data suggest that zebrafish Abcb4 functionally phenocopies P-gp and that the zebrafish may serve as a model to study the role of P-gp at the BBB.

## Introduction

The human blood–brain barrier (BBB) serves as a defense mechanism for the brain, protecting it from toxins that might gain entry via the blood circulating in the cerebral vasculature^1^. The BBB is comprised of a physical barrier in the form of endothelial cells that form tight junctions to prevent paracellular transport as well as an active barrier comprised of ATP-binding cassette (ABC) transporters expressed on the apical side of endothelial cells to redirect toxins back into the bloodstream^1,2^. Two transporters that are highly expressed at the BBB are P-glycoprotein (P-gp, encoded by the *ABCB1* gene) and ABCG2 (encoded by the *ABCG2* gene), both of which are associated with multidrug-resistant cancer^3^. The BBB is therefore a significant barrier to the successful treatment of brain malignancies and metastases, as cytotoxic substances, including targeted therapies, are transported by one or both transporters^3,4^.

Mouse models have demonstrated the significant and sometimes synergistic role of P-gp and ABCG2 in limiting brain penetration of various compounds. The importance of P-gp at the BBB was demonstrated by Schinkel and colleagues, who discovered serendipitously that mice lacking Abcb1a and Abcb1b (the mouse homologs of human ABCB1) were extremely sensitive to the anthelmintic agent ivermectin^5^. Brain levels of ivermectin were 100-fold higher in knockout mice compared to their wild-type counterparts and mice lacking P-gp were also found to have threefold higher levels of vincristine in the brain^5^. The development of mice deficient in Abcb1a/b as well as Abcg2 demonstrated a role for the transporters in keeping cytotoxins out of the brain. In the case of the mutant BRAF inhibitor encorafenib, mice lacking Abcb1a and Abcb1b demonstrated a 3.4-fold increase in brain levels; mice lacking Abcg2 had 1.8-fold higher levels and mice deficient in Abcb1a/b and Abcg2 had 16.1-fold higher brain levels 2 h after IV administration compared to wild-type mice^6^. The tight junctions formed by the endothelial cells at the BBB limit paracellular transport and augment the effects of transporters such as P-gp and ABCG2, thus resulting in the observed apparent cooperativity of the transporters^7,8^. This cooperativity has been demonstrated for several small molecule therapies and targeted therapies, including erlotinib and mitoxantrone^3,8^. While mouse models have provided valuable information regarding the role of P-gp and ABCG2 at the BBB, they are expensive to maintain and are not amenable to high-throughput screening or direct imaging of the CNS.

The zebrafish (*Danio rerio*, Dr) has emerged as a potential model for studying the role of transporters at the BBB^9–11^. Like higher vertebrate organisms, zebrafish have a BBB comprised of endothelial cells that form tight junctions characterized by expression of claudin-5 and zona occludens-1 as well as decreased transcytosis^12–14^. Zebrafish have no single direct homolog of *ABCB1*, but instead express two genes with similar characteristics, *abcb4* and *abcb5*^15,16^. It has been reported that the C219 antibody that recognizes human P-gp cross-reacts with both zebrafish Abcb4 and Abcb5^13^. Using this antibody, a protein similar to P-gp was purported to be expressed on endothelial cells that form the zebrafish brain vasculature^9,13^. However, which of the two proteins (Abcb4 or Abcb5) was expressed at the BBB was unknown. Preliminary studies demonstrated that transporters expressed at the zebrafish BBB can efflux known P-gp substrates such as rhodamine 123, loperamide and some tyrosine kinase inhibitors^11,17^. Both Abcb4 and Abcb5 have been shown to transport some substrates of human P-gp^15,18,19^; however, detailed substrate specificity testing of the two transporters has not been performed. Here, we provide a detailed characterization of the zebrafish homologs of human *ABCB1*. Additionally, we localize them to various barrier sites in the fish, suggesting that the zebrafish could indeed be an effective model to study the role of transporters at the human BBB and other sites.

## Results

### Establishment and characterization of HEK293 cells stably expressing zebrafish Abcb4 or Abcb5

**Figure 1:**
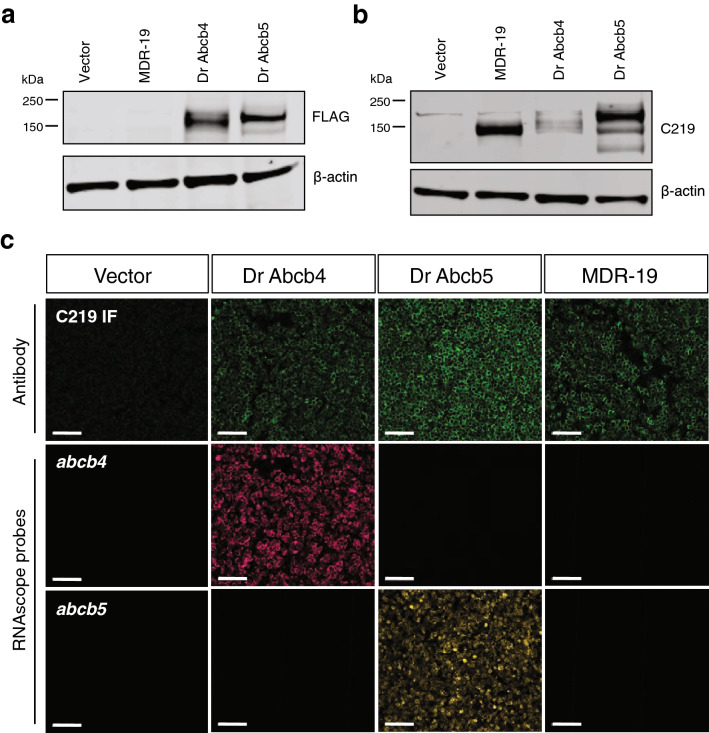
Characterization of cells transfected to express human ABCB1, zebrafish Abcb4 or Abcb5. Whole cell lysates were prepared from HEK293 cells transfected with empty vector (Vector), human ABCB1 (MDR-19), zebrafish abcb4 (Dr Abcb4) or abcb5 (Dr Abcb5), subjected to polyacrylamide gel electrophoresis and transferred to nitrocellulose. Blots were probed with anti-FLAG antibody and beta actin (**a**) or anti-ABCB1 antibody C219 and beta-actin (**b**). Sections in **(a)** were cropped from the blot in Supplementary Fig. S1a; sections in **(b)** were cropped from the blot in Supplemental Fig. S1b. (**c**) Paraffin-embedded cells (from **a** and **b**) were probed with the C219 antibody (top row, green staining) or RNAscope probes to detect zebrafish *abcb4* (middle row, magenta staining) or zebrafish *abcb5* (bottom row, yellow staining), as outlined in Materials and Methods. Bar = 100 µm.

To characterize the substrate specificity of the zebrafish homologs of human *ABCB1*, we transfected HEK293 cells with either empty vector plasmid, or vector encoding full-length *abcb4* or *abcb5*, with both proteins having a 5' FLAG tag. Clones were selected based on reactivity with an anti-FLAG antibody, and we chose two clones with similar levels of expression for further study, Dr Abcb4 and Dr Abcb5, as shown in Fig. 1a (complete blots are provided in Fig. S1). The predicted molecular weight of zebrafish Abcb4 is approximately 141 kDa and Abcb5 is 147 kDa and the FLAG-tagged proteins ran slightly higher. The C219 antibody that detects human P-gp has been reported to react with both zebrafish Abcb4 and Abcb5^13^, but this has not been validated with each protein separately. Interestingly, despite the findings with the anti-FLAG antibody, the C219 antibody seemed to preferentially stain Abcb5 over Abcb4 by immunoblot (Fig. 1b). The MDR-19 cell line, derived from HEK293 cells transfected to overexpress human P-gp, served as a positive control for C219 staining. Assuming the affinity of the C219 antibody is similar for Dr Abcb5 and P-gp, the immunoblot analyses suggest similar levels of transporter expression in all of the lines transfected to express the transporters.

Transporter expression was measured by immunofluorescence with the C219 antibody and expression of zebrafish Abcb4 and Abcb5 at the cell membrane was noted on both of the selected clones, with slightly more staining of the Abcb5 clone (Fig. 1c, top row). Expression of P-gp in the MDR-19 cell line was also similar to expression of zebrafish Abcb4 and Abcb5 in the transfected lines. The specificity of the RNA in situ probes was validated in the transfected cell lines and we found that only the Dr Abcb4 cells stained positively with the *abcb4* probe (Fig. 1c, middle row), while only the Dr Abcb5 cells stained positively with the *abcb5* probe (Fig. 1c, bottom row); empty vector and MDR-19 cells were not found to react with either probe.

### Cytotoxicity assays demonstrate zebrafish Abcb4 is more similar to human P-gp than zebrafish Abcb5

Having confirmed that our clones all expressed similar levels of zebrafish Abcb4, Abcb5 or human P-gp, we performed 3-day cytotoxicity assays with known substrates of human P-gp: vinblastine, doxorubicin, bisantrene, etoposide, mitoxantrone and paclitaxel. Camptothecin was chosen as a negative control, as P-gp overexpression does not confer resistance to that particular drug. As shown in Fig. S2, overexpression of zebrafish Abcb4 or Abcb5 conferred resistance to etoposide, paclitaxel, bisantrene, and vinblastine. Abcb4 clearly conferred greater resistance to bisantrene, mitoxantrone, and doxorubicin compared to Abcb5. No cross resistance to camptothecin was observed in any of the cells; GI_50_ values are summarized for the compounds in Table S1. These initial studies suggested that zebrafish Abcb4 and Abcb5 have slightly different substrate specificities and that zebrafish Abcb4 may be functionally similar to human P-gp.

### High throughput screening of known P-gp substrates

**Figure 2:**
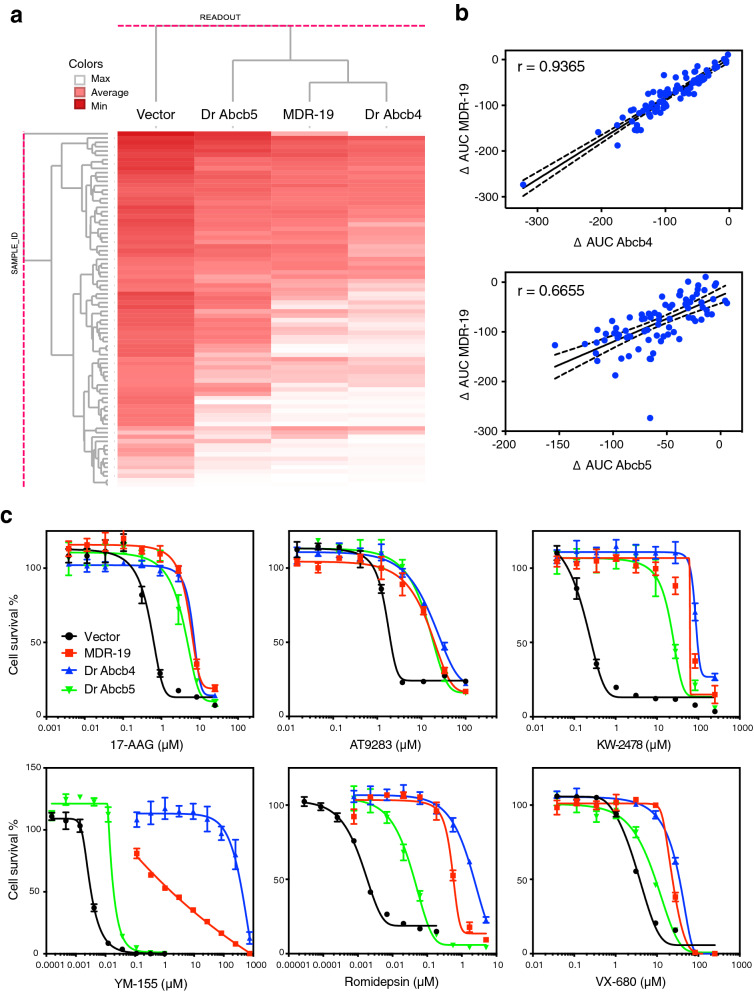
High-throughput screening demonstrates that substrate specificity of zebrafish Abcb4 closely resembles that of human P-gp. (**a**) High throughput screening was performed with 90 human P-gp substrates with empty vector transfected cells (Vector) or cells transfected to express zebrafish Abcb4 (Dr Abcb4), zebrafish Abcb5 (Dr Abcb5) or human P-gp (MDR-19). Compound activity was then subjected to unsupervised clustering against Vector, Dr Abcb4, Dr Abcb5 and MDR-19 cells. Darker red color represents greater cytotoxicity. (**b**) The difference in AUC (delta AUC) was determined as the difference between Vector cells as compared to Dr Abcb4, Dr Abcb5 or MDR-19 cells. The delta AUC values for human P-gp (MDR-19 cells) were compared to those of zebrafish Abcb4 (Dr Abcb4) or zebrafish Abcb5 (Dr Abcb5). Solid lines represent lines of best fit and dashed lines are 95% confidence intervals. (**c**) Three-day cytotoxicity assays were performed with 6 substrates included in the screen—17-AAG, AT9283, KW-2478, romidepsin, VX-680 and YM-155. Results from all the experiments are summarized in Table S2.

We recently described the development of a high throughput screen to identify substrates of human P-gp^20^. Here, we compared the substrate specificity of zebrafish Abcb4 and Abcb5 to human P-gp by calculating area under the curve (AUC) values for dose–response curves generated from the transfected cells that were tested with of a panel of 90 cytotoxic human P-gp substrates^20^. Results from the screen are shown in the heat map in Fig. 2a. Unsupervised clustering demonstrated that Abcb4 was functionally similar to human P-gp. As seen in Fig. 2b, the AUC values for zebrafish Abcb4 correlated better with human P-gp (r = 0.94) than with Abcb5 (r = 0.67). Data for each of the cell lines tested with the panel of 90 cytotoxic human P-gp substrates was deposited in PubChem with AID 1,508,636 for MDR-19 cells (https://pubchem.ncbi.nlm.nih.gov/bioassay/1508636), 1,508,637 for empty vector cells (https://pubchem.ncbi.nlm.nih.gov/bioassay/1508637), 1,508,635 for Dr Abcb4 cells (https://pubchem.ncbi.nlm.nih.gov/bioassay/1508635) and 1,508,633 for Dr Abcb5 cells (https://pubchem.ncbi.nlm.nih.gov/bioassay/1508633).

Of the 90 substrates that were tested, 6 were subsequently selected to confirm the results of the high-throughput screen; three for which AUC values were similar in all three transporters—17-AAG (HSP90 inhibitor), AT9283 (Janus kinase/aurora kinase inhibitor), and KW-2478 (HSP90 inhibitor)—and three for which AUC values for Abcb5 were lower than those for Abcb4 or P-gp—YM-155 (survivin inhibitor), romidepsin (histone deacetylase inhibitor), and VX-680 (tozasertib, aurora kinase inhibitor). We performed confirmatory three-day cytotoxicity assays on empty vector transfected cells or cells transfected to express Dr Abcb4, Dr Abcb5, or human P-gp. Comparing the cytotoxicity profiles of the 6 substrates in the transporter-expressing HEK cells, we found that resistance to 17-AAG, AT9283 and KW-2478 was similar in the three transporter-expressing cell lines (Fig. 2c). However, for some compounds, Abcb4 was clearly the better transporter, most notably for YM-155, as zebrafish Abcb5 conferred low levels of resistance to that compound compared to zebrafish Abcb4 or human P-gp (Fig. 2c). Data for the 6 compounds are summarized in Table S2. We conclude that in terms of substrate specificity, zebrafish Abcb4 is more like human P-gp and that zebrafish Abcb5, while functional, has a slightly narrower substrate specificity compared to Abcb4.

### Efficacy of zebrafish P-gp homolog inhibition is inhibitor- and substrate-dependent

**Figure 3:**
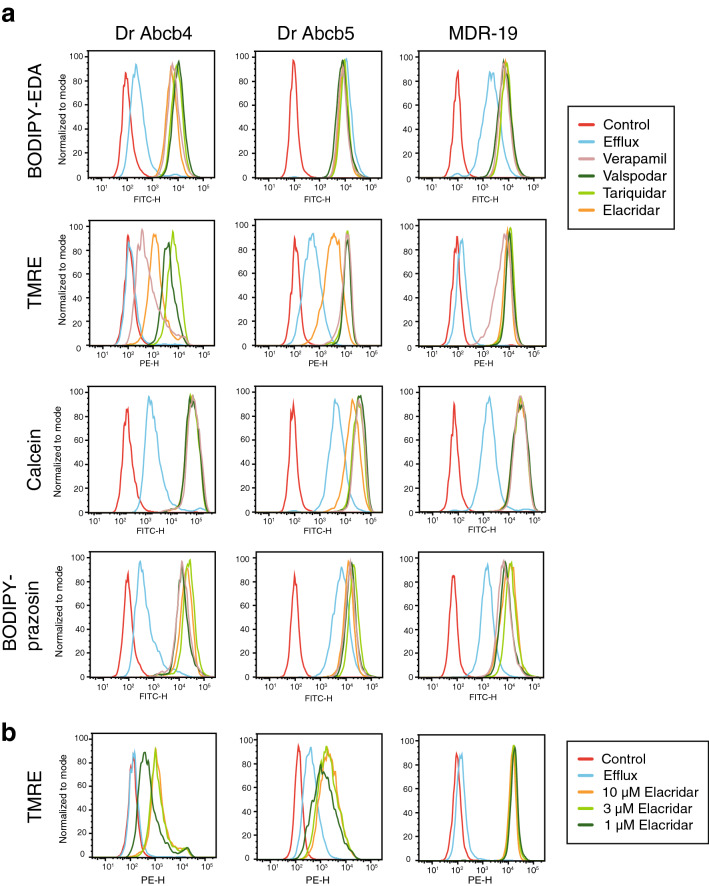
Zebrafish Abcb4 and Abcb5 differentially transport fluorescent P-gp substrates. (**a**) HEK293 cells transfected to express zebrafish Abcb4 (Dr Abcb4), zebrafish Abcb5 (Dr Abcb5) or human P-gp (MDR-19) were incubated in medium with 0.5 µM BODIPY FL-EDA, 0.5 µM TMRE, 150 nM calcein-AM or 0.5 µM BODIPY prazosin in the presence or absence of 10 µM elacridar, 10 µM tariquidar, 10 µM valspodar, or 100 µM verapamil for 30 min. The medium was removed and replaced with substrate-free medium in the presence or absence of inhibitor for an additional 1 h. Cells were incubated with fluorescent substrate alone, yielding the Efflux histogram and cell autofluorescence is depicted by the Control histogram. (**b**) Cells from (**a**) were incubated with medium containing 0.5 µM TMRE in the presence or absence of 1, 3, or 10 µM elacridar for 30 min after which the medium was removed and replaced with substrate-free medium in the presence or absence of inhibitor for an additional 1 h.

We next compared the ability of human P-gp and zebrafish Abcb4 and Abcb5 to transport known fluorescent substrates of human P-gp using flow cytometry. We also examined the ability of some common P-gp inhibitors to abrogate transporter activity, as P-gp inhibitors are frequently used as inhibitors of zebrafish Abcb4 and Abcb5^11,15,18^, despite the fact that their efficacy with different substrates has not been carefully examined. The substrates tested included calcein AM, rhodamine 123, BODIPY-prazosin, Flutax, BODIPY-vinblastine, LDS 751, BODIPY EDA, and TMRE. The human P-gp inhibitors examined were elacridar (10 µM), valspodar (10 µM), tariquidar (10 µM), and verapamil (100 µM). Cells were incubated with fluorescent substrate alone (Efflux histogram) or in the presence of inhibitors; cell autofluorescence is depicted by the Control histogram. In the case of MDR-19 cells that overexpress human P-gp (Fig. 3a and Fig. S3), all the fluorescent probes were transported, as fluorescence of cells incubated with the substrate alone (Efflux, blue histogram) was lower compared to cells co-incubated with probe and elacridar (orange), tariquidar (light green), valspodar (dark green) or verapamil (pink). Fluorescence histograms of probes co-incubated with inhibitor were overlapping, suggesting complete P-gp inhibition by the various inhibitors.

We found that zebrafish transporter substrates could be grouped into three categories. First, BODIPY-EDA (Fig. 3a, top row) and LDS 751 (Fig. S3, top row) were transported by Abcb4, but not Abcb5. The intracellular fluorescence of these substrates was decreased in cells expressing Abcb4, as shown by the blue histogram, whereas this histogram is closely aligned with the inhibitor histograms in the case of the Abcb5-overexpressing cells, signifying no transport. Second, although some substrates were transported by both Abcb4 and Abcb5, human P-gp inhibitors did not fully inhibit the zebrafish transporters. This was the case for TMRE (Fig. 3a, second row) and rhodamine 123 (Fig. S3, second row), most likely due to their similar chemical structures. In the case of Abcb4 and TMRE, both the verapamil (pink) and elacridar (orange) histograms appeared to the left of the tariquidar (light green) and valspodar (dark green) histograms, denoting incomplete inhibition of transport. A similar result was observed with Abcb5 and TMRE, although only elacridar (orange) appeared to the left of histograms for the other inhibitors. Elacridar, verapamil and tariquidar were less effective than valspodar at inhibiting Abcb4-mediated rhodamine transport, while elacridar was less effective at inhibiting Abcb5-mediated rhodamine transport. The other substrates—calcein AM and BODIPY-prazosin (Fig. 2a third and fourth row) as well as Flutax, and BODIPY-vinblastine (Fig. S3, third and fourth row)—fell into the third category, where all appeared to be transported and all inhibitors seemed to be equally effective at inhibiting the zebrafish transporters.

To compare the ability of elacridar to inhibit the three transporters, we performed a dose response with TMRE as the substrate since elacridar was unable to completely inhibit TMRE efflux from cells expressing zebrafish Abcb4 or Abcb5. In the case of P-gp, elacridar at the lowest concentration of 1 µM (dark green histogram) completely inhibited P-gp-mediated TMRE efflux (Fig. 3b). In the case of zebrafish Abcb4 or Abcb5, even 10 µM was unable to completely inhibit TMRE transport. Thus, known P-gp inhibitors might not be effective at inhibiting zebrafish Abcb4 or Abcb5 even at relatively high concentrations with some substrates.

### ATPase activity of zebrafish homologs of human ABCB1

**Figure 4:**
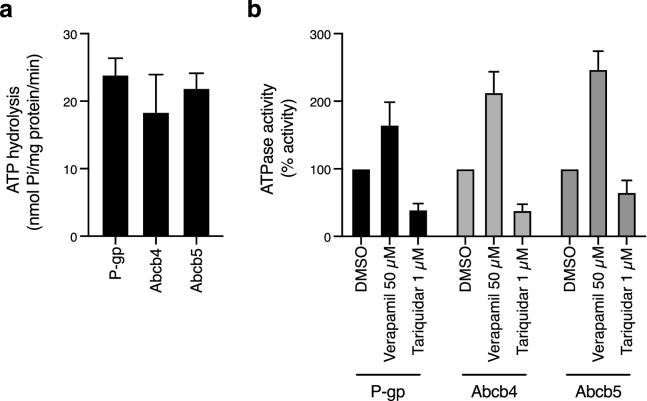
ATPase activity of zebrafish Abcb4 and Abcb5 is similar to that of human P-gp. (**a**) The vanadate-sensitive activity of zebrafish Abcb4, zebrafish Abcb5 and human P-gp was determined as outlined in Materials and Methods using membranes isolated from Dr Abcb4, Dr Abcb5 or MDR-19 cells, respectively. (**b**) Basal P-gp ATPase activity (DMSO) was compared to activity in the presence of 50 µM verapamil, or in the presence of 1 µM tariquidar. Graphs from (**a**) and (**b**) depict average values from three independent experiments (error bars ± SD).

Verapamil is both a substrate and inhibitor of P-gp and induces ATPase activity that is coupled to drug transport^21^. Tariquidar is a P-gp inhibitor that is not itself transported by P-gp and is known to decrease ATPase activity of P-gp due to high affinity interactions^22^. We thus compared the verapamil-stimulated ATPase activity of human P-gp to zebrafish Abcb4 and Abcb5. As shown in Fig. 4a, basal levels of ATPase activity were similar among P-gp, zebrafish Abcb4 and Abcb5. For all proteins, verapamil significantly stimulated ATPase activity compared to basal levels (*p* < 0.02). This is in agreement with a previous report in which verapamil-inducible ATPase activity was demonstrated for zebrafish Abcb4^15^ as well as more recently for yellowfish tuna ABCB1^23^. Basal ATPase activity could be inhibited by the addition of 1 µM tariquidar (Fig. 4b); however, the effect was not significant in the case of zebrafish Abcb5, possibly due to structural differences between P-gp and Abcb5.

### Molecular modeling of zebrafish Abcb4 and Abcb5 reveals potential differences in the drug-binding pocket

**Figure 5:**
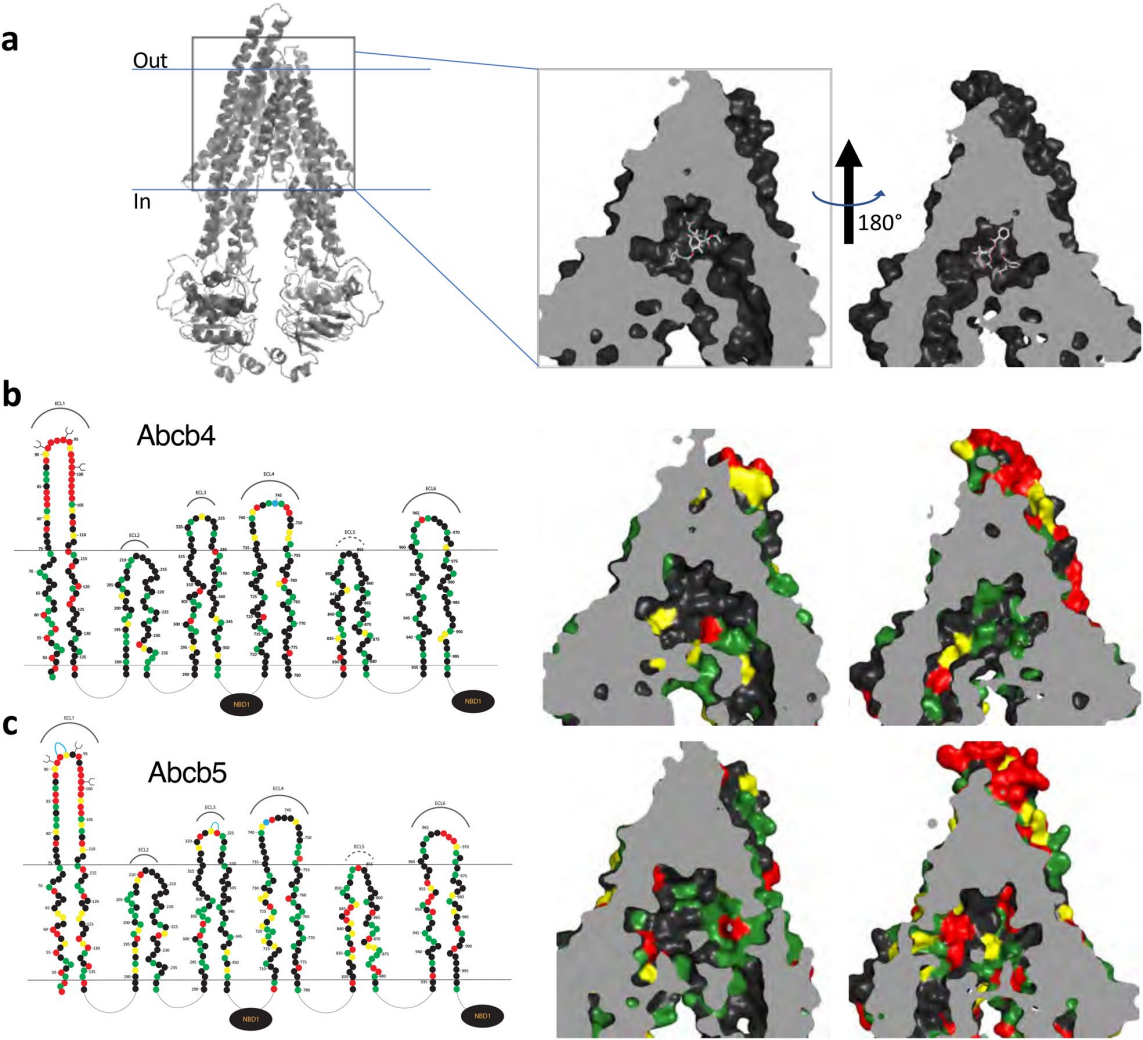
3D homology modeling of amino acid similarity in the binding pocket of zebrafish Abcb4 and Abcb5. (**a**) 3D modeling of the human P-gp structure that models P-gp in complex with taxol and the Fab of the UIC2 antibody (PDB:6QEX). The model is split into two halves to provide a clearer view. Amino acid differences in the transmembrane regions of the models of human P-gp and zebrafish Abcb4 (**b**) and Abcb5 (**c**) are shown. In (**b**) and (**c**), on the left side linear 2D models of Abcb4 and Abcb5 are shown. Amino acids (filled circles) similar to human P-gp are shown in green (most similar), yellow (fairly similar) or red (least similar).

To look for potential explanations for the differences in substrate and inhibitor specificity between P-gp and zebrafish Abcb4 and Abcb5, homology models of each protein were generated using the Swiss-model program (https://swissmodel.expasy.org/). The human P-gp structure PDB:6QEX was used as a template, which corresponds to P-gp in complex with taxol and the Fab of the UIC2 antibody^24^. The Clustal Omega program (https://www.ebi.ac.uk/Tools/msa/clustalo/) was used to align the sequences relative to human P-gp. Figure 5a shows the binding pocket of human P-gp with taxol bound at the center of the cavity. The model is split into two halves to provide a clearer view of both sides of the molecule. We found 64% and 58% amino acid identity between P-gp and zebrafish Abcb4 and Abcb5, respectively. The differences between the amino acids in the transmembrane region between P-gp and zebrafish Abcb4 (Fig. 5b) and Abcb5 (Fig. 5c) were then interrogated. Amino acids were color-coded based on their similarity to the corresponding amino acid in human P-gp with identical amino acids represented in black and different amino acids colored green (most similar) to yellow (fairly similar) to red (least similar). Both proteins showed differences with human P-gp in the drug binding pocket; however, we noted more black and green residues in the binding pocket of Abcb4 compared to P-gp, while more red and yellow residues were noted in the binding pocket of Abcb5. These structural differences in the residues potentially explain the difference in substrate specificity between the proteins.

### Immunohistochemical localization of Abcb4 and Abcb5 in adult zebrafish

**Figure 6:**
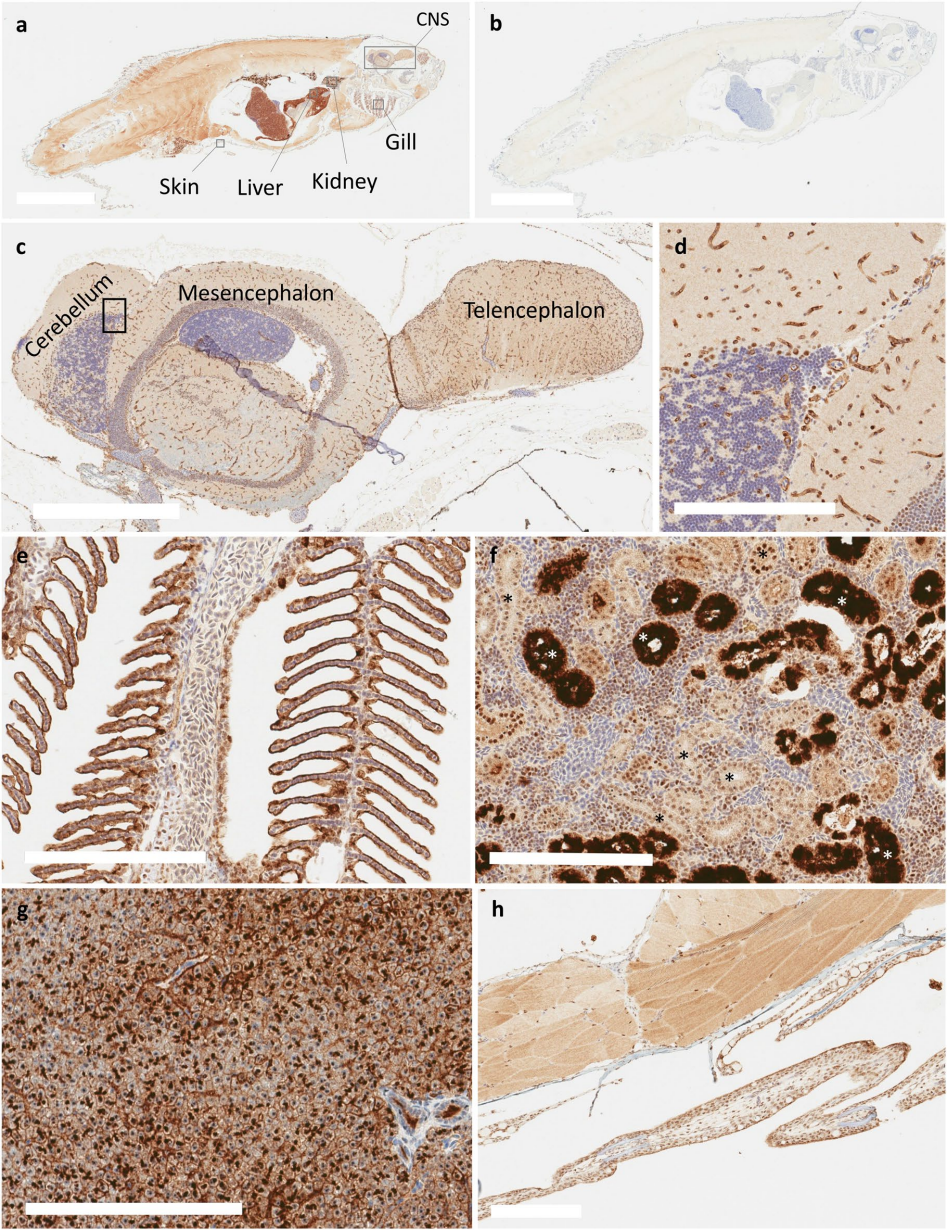
Immunohistochemical staining of Abcb4 and Abcb5 with the C219 antibody in adult zebrafish. Whole adult zebrafish were stained with the C219 antibody (**a**) or negative control (**b**), as noted in Materials and Methods. Bar = 5 mm for (**a**) and (**b**). Regions of interest are indicated (**a**) and expanded in subsequent panels (**c–h**). C219 staining of the zebrafish brain (**c**) with an enlarged portion of the cerebellum (**d**). Positive staining of C219 antibody was also noted in gill epithelium (**e**), a subset of renal tubules or collecting ducts (*) (**f**), hepatocytes (**g**), and the epidermis (**h**). Bar = 700 µm for (**c**) and 200 µm for (**d–h**).

In light of the slightly different substrate specificities of the two different homologs, we decided that it was important to localize the transporters in the zebrafish to determine whether the zebrafish could be used as a model to study the human blood–brain barrier. We first performed immunohistochemistry analysis of the zebrafish P-gp isoforms using the C219 antibody. Staining of the whole fish is shown in Fig. 6a, with the negative control shown in Fig. 6b. Specific areas of positivity were then examined as denoted by the boxed areas in Fig. 6a. We found C219 reactivity in the vasculature of the brain, including the cerebellum, mesencephalon and telencephalon (Fig. 6c). Higher magnification of the cerebellum revealed staining consistent with brain vasculature (Fig. 6d). Staining was also noted in the gill epithelium (Fig. 6e), kidney (Fig. 6f), hepatocytes (Fig. 6g) and skin (Fig. 6h). This expression pattern is similar to that of humans, where P-gp is expressed in the BBB, liver, and the kidneys^25^.

### Organ-specific expression patterns are observed for zebrafish abcb4 and abcb5

**Figure 7:**
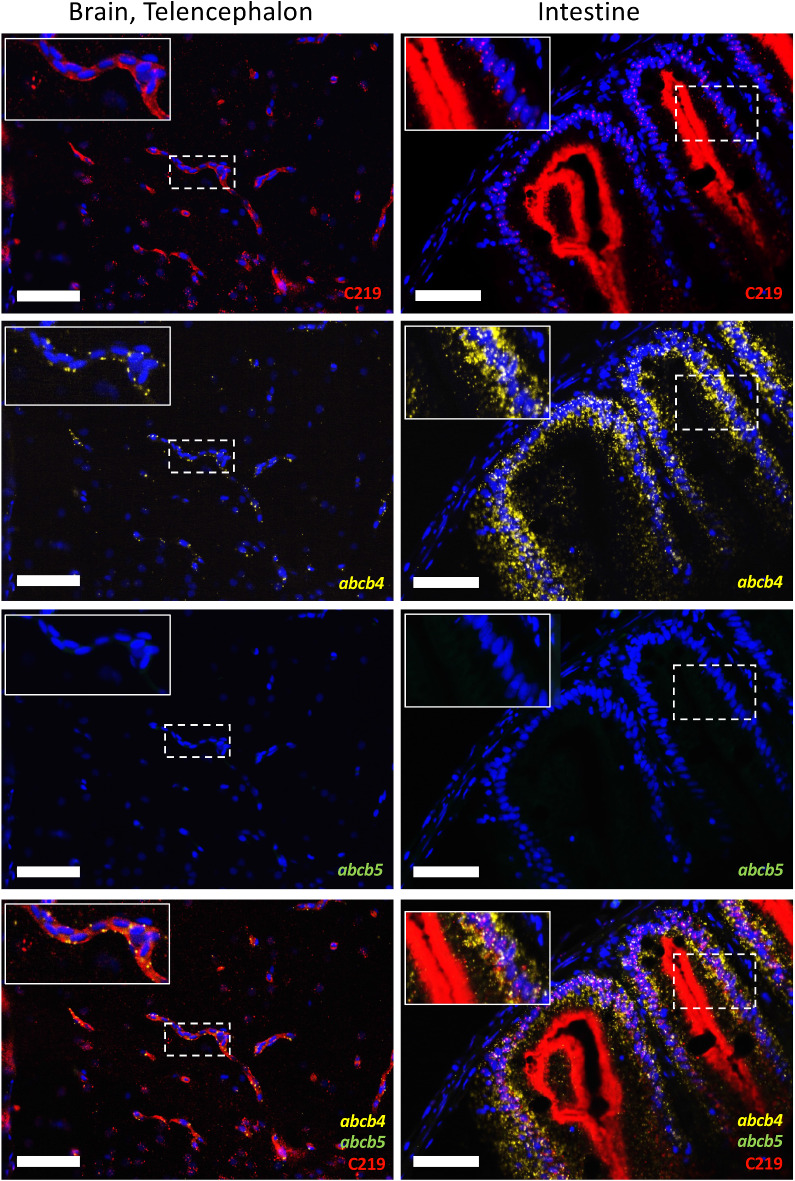
Zebrafish abcb4 RNA colocalizes with C219 staining in the zebrafish brain (telencephalon) and gastrointestinal tract of adult zebrafish. Zebrafish sections were stained with RNAscope abcb4 (yellow) and abcb5 (green) probes followed by the C219 antibody (red), as outlined in Materials and Methods. Fluorescence channels were interrogated individually and merged in sections of the zebrafish brain (left column) or gastrointestinal tract (right column). Bars = 50 µm. Nuclei were stained with DAPI (blue). Inset shows an enlarged portion of each image, denoted by the dashed box.

As the C219 antibody cannot qualitatively differentiate between the two zebrafish isoforms, as demonstrated in Fig. 1c, we coupled antibody staining with RNAscope technology that recognizes RNA expression. In the brain microvasculature, we found that the C219 antibody (red) colocalized with the probe for *abcb4* (yellow), while the *abcb5* probe (green) was not detected (Fig. 7, left column). Inset images are magnifications of the areas denoted by the dashed boxes. Similar results were observed in the gastrointestinal tract, where *abcb4* (yellow) was colocalized with the C219 antibody (red), but *abcb5* (green) was not detected (Fig. 7, right column). Interestingly, high levels of predominantly *abcb5* were observed in earlier follicular stages of the ovary but not in later stages (Fig. S4). In the liver, we noted that expression of *abcb4* was predominant. High levels of C219 staining (red) were observed in the renal tubules of the kidney and distinct regions of the nephron stained positive for either *abcb4* (yellow) or *abcb5* (green, Fig. S4). The expression patterns of abcb4 and abcb5 in various organs are summarized in Table S3 and representative images are provided in Fig. S5 for each of the organs examined.

**Figure 8:**
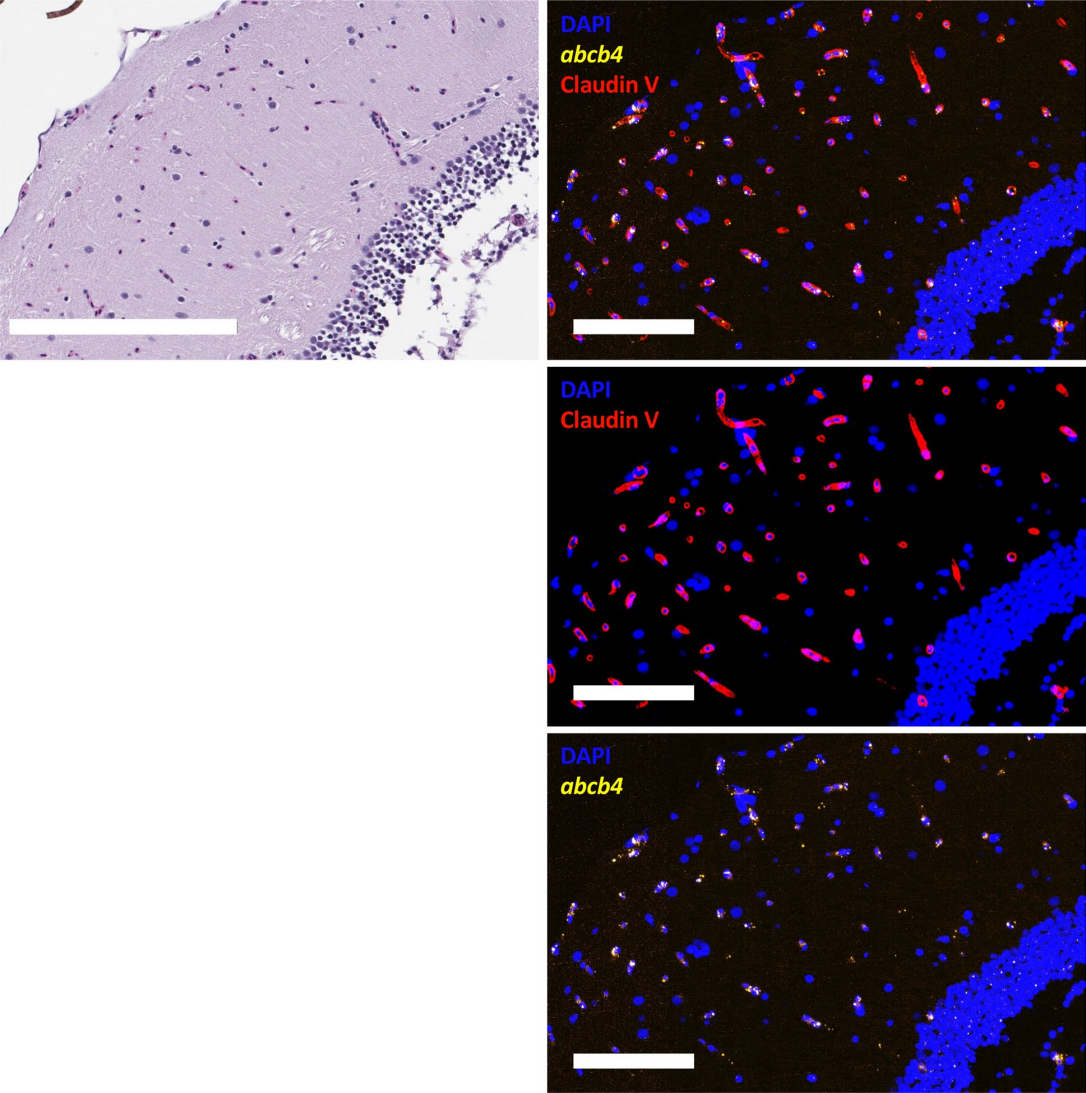
Zebrafish abcb4 colocalizes with claudin-5 in the zebrafish brain (mesencephalon). Zebrafish sections were stained with H&E and RNAscope abcb4-C2 (green) followed by an antibody to claudin-5 (red), as outlined in Materials and Methods. Nuclei were stained with DAPI (blue). Fluorescence channels were interrogated individually and merged. Bar = 200 µm for H&E image and 100 µm for fluorescence images.

To confirm Abcb4 expression in the brain vasculature, we stained for the tight junction protein claudin-5 (red) in the mesencephalon and combined this with the *abcb4* probe (yellow, Fig. 8). Our staining indicates that *abcb4* colocalizes with the Claudin V throughout the CNS, including the vasculature in neuroparenchyma adjacent to the ventricular system. Zebrafish Abcb4, with a substrate specificity that phenocopies human P-gp, appears to be the only *ABCB1* isoform in the brain vasculature of the zebrafish.

## Discussion

The zebrafish is emerging as a suitable model for studying the human BBB, and with the development of oral dosing techniques, it may also be useful in modeling the oral bioavailability of drugs^26^. While mammals are known to express ABC transporters at the BBB and in the gastrointestinal tract, the localization and substrate specificity of homologous transporters in the zebrafish had not been carefully studied. Here, we report that zebrafish Abcb4 is the predominant transporter expressed at the zebrafish BBB and intestine, that both transporters are expressed in various regions of the kidney, and that Abcb5 is nearly exclusively expressed in the gill, skin, and ovary. Additionally, in terms of substrate specificity, zebrafish Abcb4 is functionally similar to human P-gp, suggesting that zebrafish *abcb4* is actually the homolog to human *ABCB1*. Based on transporter localization and specificity, the zebrafish may represent a valuable model for studying the oral bioavailability of compounds and the uptake of drugs into the brain.

Like the mammalian BBB, the endothelial cells of the zebrafish BBB form tight junctions characterized by expression of ZO-1 and claudin-5 and are in close contact with pericytes^27^. However, zebrafish appear to lack homologous astrocytic foot processes at the BBB and instead have radial glia cells that do not sheathe the endothelial cells to the extent observed in mammals, leaving their function at the BBB unclear^27,28^. While previous reports have suggested that a homolog of human P-gp is expressed at the zebrafish BBB, this was concluded based on reactivity with the anti-human P-gp antibody C219, which was purported to recognize both zebrafish homologs of *ABCB1*^9,13^. Consistent with these previous reports, we also found C219 reactivity in the endothelial cells of the zebrafish brain, and using RNA in situ techniques, have identified *abcb4* as the gene responsible for the observed C219 staining. Thus, the zebrafish could indeed be a powerful tool to study the role of transporters in limiting the brain penetration of drugs, and initial studies have shown that the zebrafish could be comparable to mouse models to determine whether drugs can cross the BBB^11^.

Concerning the two zebrafish proteins that are similar to human P-gp, we find that both zebrafish Abcb4 and Abcb5 transport known P-gp substrates, but that Abcb5 has a slightly narrower substrate specificity profile compared to Abcb4 based on our studies with cytotoxic agents and known fluorescent P-gp substrates. Previous work by Fischer and colleagues found that Abcb4 could transport several fluorescent P-gp substrates, but they suggested that Abcb5 might not be a xenobiotic transporter based on morpholino knockdown of *abcb5* in zebrafish embryos^15^. More recently, Gordon et al. reported efflux of fluorescent P-gp substrates in zebrafish ionocytes and found this to be due to overexpression of *abcb5*^18^. Our work expands on these previous reports, as we show that both zebrafish Abcb4 and Abcb5 are functional transporters. The zebrafish Abcb4 protein was shown to transport all fluorescent P-gp substrates tested and was found to confer resistance to 90 P-gp substrates examined by high-throughput screening, while zebrafish Abcb5 conferred resistance to a slightly narrower range of substrates. However, we also noted some differences in inhibitor specificity that were transporter- and substrate-dependent. Care must therefore be taken when evaluating the role of transporter inhibitors in modulating Abcb4 in order to increase brain penetration. Additionally, we would like to point out that our conclusions concerning substrate specificity are based on results obtained with zebrafish proteins expressed in mammalian cells. While this allows for high-throughput assays, it is possible that the zebrafish proteins behave differently in vivo.

Although techniques for oral dosing of zebrafish have been developed^26^, little is known in terms of the transporters expressed in the gastrointestinal tract. However, expression of *ABCB1* homologs has been described in other fish species^16^, suggesting that this may also be true for the zebrafish. We found high reactivity with the C219 antibody in the gastrointestinal tract of the zebrafish and found that this was mainly due to high levels of *abcb4* expression. This is in agreement with Umans and Taylor, who reported C219 staining in the intestinal epithelium of zebrafish larvae^9^ and Lu et al., who found high levels of *abcb4* gene expression in the intestine of adult zebrafish^29^. The high levels of Abcb4 in the zebrafish gut appear to suggest that the zebrafish could be used in pre-clinical studies to determine the oral bioavailability of drugs. But, as mentioned above, the role that inhibitors might play to increase oral bioavailability would need to be scrutinized, given the variable effects of P-gp inhibitors.

One lingering question regarding transporters expressed at the zebrafish BBB and in the gut is the involvement of *ABCG2* homologs. Zebrafish have four direct *ABCG2* homologs—*abcg2a*, *abcg2b*, *abcg2c* and *abcg2d*—and little is known about their expression or substrate specificity. In one of the most comprehensive studies of these homologs, Kobayashi and colleagues found overexpression of zebrafish *abcg2a* and *abcg2c* in side-population cells isolated from the kidney, which is the main organ of hematopoiesis in the zebrafish^30^. Additionally, they reported expression of *abcg2a*, *abcg2b,* and *abcg2c* in the intestine^30^. Although cells transfected to overexpress zebrafish Abcg2a were found to transport Hoechst 33342, this appears to be the only known substrate of the zebrafish ABCG2 homologs^30^. Further work is needed to characterize these proteins and determine their potential role in the gut and BBB.

Our findings that abcb4 and abcb5 are expressed at specific sites raises multiple questions. Why are there two transporters that have a similar function? Are they expressed at specific sites for specific reasons? In this work, we noted high levels of Abcb5 in the ovaries. Is there a particular substrate that Abcb5 can transport that Abcb4 cannot transport, thus making Abcb5 better suited to this site? We did note the differences in amino acids in Abcb4 and Abcb5, especially in the drug binding pocket. Interestingly, it has been suggested that the evolution of substrate specificity results primarily from either mutations in the transmembrane domain where drug binding occurs, or mutations near the helices that connect the nucleotide binding domains^31^.

In summary, we find that expression of zebrafish Abcb4 at the zebrafish BBB phenocopies the role of human P-gp at the human BBB, suggesting that zebrafish Abcb4 could be considered a true homolog of P-gp. Although questions remain in terms of other transporters such as ABCG2, the zebrafish may be a powerful tool to study the role of transporters at barrier sites.

## Materials and methods

### Chemicals

Bisantrene, doxorubicin, mitoxantrone, paclitaxel, rhodamine 123, vinblastine and verapamil were purchased from Sigma-Aldrich (St. Louis, MO). BODIPY FL-ethylenediamine (EDA), BODIPY-prazosin, BODIPY-vinblastine, calcein-AM, and tetramethylrhodamine ethyl ester (TMRE) were obtained from Invitrogen/Life Technologies (Carlsbad, CA). Laser dye styryl 751 (LDS 751) was purchased from Santa Cruz Biotechnology (Dallas, TX). Flutax was from Tocris Bioscience (Minneapolis, MN). AT9283, KW2478 and valspodar were from Apex Biotechnology (Houston, TX). YM-155, VX-680, and 17-AAG were purchased from ChemieTek (Indianapolis, IN). Romidepsin was obtained from Selleck Chem (Houston, TX). Elacridar and tariquidar were from MedChemExpress (Monmouth Junction, NJ).

### Zebrafish husbandry

Animal studies were conducted under a protocol approved by the National Cancer Institute-Bethesda Animal Care and Use Committee (IACUC Number LCB-033-A). Zebrafish (TAB-5 strain) were maintained at 28.5 °C on a 14-h light/10-h dark cycle according to standard procedures as previously described^32^. Adult fish were obtained from larvae generated by natural spawning, raised at 28.5 °C, and maintained in fish water (60 mg Instant Ocean^©^ sea salt [Instant Ocean, Blacksburg, VA] per liter of DI water). Water was changed daily and fish were checked regularly for development. Feeding began at 5 days post fertilization (dpf). All experiments were conducted in accordance with policies and procedures set forth by the NCI-Bethesda Animal Care and Use Committee.

### Cell culture

HEK293 cells (ATCC, Manassas, VA) were transfected with empty pcDNA3.1 vector or with vector containing full-length *abcb4* or *abcb5* (all purchased from Genscript, Piscataway, NJ) flanked by a FLAG tag. Sequences were verified before transfection. Transfected cells were grown in MEM medium (Mediatech, Manassas, VA) and were maintained in 2 mg/ml G418 (Mediatech). Clones expressing similar levels of Abcb4 or Abcb5 protein were selected based on FLAG expression as detected by immunoblot. The MDR-19 cell line was generated from HEK293 cells transfected with full-length human *ABCB1* and has been previously described^33^.

### Flow cytometry

Flow cytometry studies with fluorescent P-gp substrates were performed as previously described^33,34^. Briefly, trypsinized cells were incubated with the desired fluorescent substrate (150 nM calcein-AM, 5 µM Flutax, 0.5 µM BODIPY-prazosin, 250 nM BODIPY-vinblastine, 0.5 µM BODIPY EDA, 0.5 µM TMRE, 0.5 µM LDS 751, or 0.5 µg/ml rhodamine 123) in the presence or absence of the desired inhibitor (10 µM elacridar, 10 µM tariquidar, 10 µM valspodar, or 100 µM verapamil) for 30 min. Subsequently, the medium was removed and replaced with substrate-free medium in the presence or absence of inhibitor for an additional 1 h. Cells that were incubated with fluorescent substrate alone yielded the Efflux histogram, while cell autofluorescence is depicted by the Control histogram. Samples were then analyzed on a FACSCanto II flow cytometer (BD Biosciences San Jose, CA) and data were analyzed with FlowJo software (v 10.6.1, Tree Star, Inc, Ashland, OR).

### High-throughput screening

A high-throughput screen was performed as previously described^20^ on empty vector transfected cells, P-gp-overexpressing MDR-19 cells and cells transfected with full-length zebrafish *abcb4* (Dr Abcb4) or *abcb5* (Dr Abcb5). Briefly, cells were plated into 1536-well plates (Corning 7464) at a density of 500 cells/well in 5 µL media. P-gp substrate compounds (90) selected from our previous study^20^ were then added at varying concentrations using a 1536-head pin tool (Kalypsis, San Diego, CA) and plates were incubated at 37 °C in 5% CO_2_ for 72 h. CellTiter-Glo reagent (Promega) was dispensed into the wells, incubated for 5 min and luminescence was read on a ViewLux instrument (Perkin-Elmer). Data was normalized based on DMSO basal level (0% activity) and no cell control (-100% activity), which mimics the maximal cell killing effect from this assay. The area-under-the-curve (AUC) was assessed for each compound and was compared for each cell line as previously described^20^. More negative AUC values represented greater cytotoxicity. Compounds were further clustered hierarchically using TIBCO Spotfire 6.0.0 (Spotfire Inc., Cambridge, MA. https://spotfire.tibco.com/) based on AUC values from the screen.

### Immunoblot

Whole cell lysates (30 µg) were obtained from transfected cells using RIPA buffer, heated to 37° for 20 min, then subjected to electrophoresis on a premade 4–12% bis–tris gel and transferred to a nitrocellulose membrane. Subsequently the blot was blocked with Odyssey Blocking Buffer (Li-COR, Lincoln, NE) for one hour at room temperature, then probed overnight with mouse monoclonal anti-GAPDH (American Research Products, Waltham, MA, 1:8000), the anti-P-gp antibody C219 (Signet Laboratories, Dedham, MA, 1:250), and anti-FLAG antibody (Millipore-Sigma, Milwaukee, WI, 1:1000), where noted. The blot was then incubated with a goat anti-mouse secondary antibody tagged with a near infra-red fluorochrome and fluorescence was measured using the LiCor ODYSSEY CLx (Li-COR).

### Cytotoxicity assays

Trypsinized cells were counted and plated into 96-well white opaque plates (5000 cells/well) and allowed to attach overnight. Drugs were subsequently added, with each concentration tested in triplicate, and plates were incubated for 72 h at 37 °C in 5% CO_2_. CellTiter-Glo (Promega, Madison, WI) reagent was used to determine cell survival according to the manufacturer’s instructions. Luminescence was subsequently read on a Tecan Infinite M200 Pro microplate reader (Tecan Group, Morrisville, NC). GI_50_ (50% growth inhibitory) concentration values were calculated as the concentration at which 50% luminescence was observed compared to untreated cells.

### ATPase assay

ATPase assays were performed as previously described^21^. Total membranes were isolated from HEK293 cells transfected to express zebrafish *abcb4* (Dr Abcb4), *abcb5* (Dr Abcb5) or human *ABCB1* (MDR-19). Vanadate-sensitive ATPase activity was calculated by measuring the end point phosphate release in the absence and presence of vanadate. Solutions containing 4.0 µg of total membrane protein in 100 µL of ATPase assay buffer (50 mM MES-Tris pH 6.8, 50 mM KCl, 5 mM sodium azide, 1 mM EGTA, 1 mM ouabain, 2 mM DTT, 10 mM MgCl_2_) with 1% DMSO solvent alone (basal activity) or with variable concentrations (0.1, 1 or 10 µM) of the substrates in DMSO were prepared. The tubes were incubated for 3 min at 37 °C, after which the reaction was initiated by addition of 5 mM ATP. After a 20-min incubation, the reaction was stopped by addition of 2.5% SDS. The amount of inorganic phosphate released was subsequently quantified by a colorimetric assay^21^. Three independent experiments were performed, and statistical significance was determined by a one-way ANOVA with a correction for multiple comparisons.

### Immunofluorescence and RNAscope probes

Adult zebrafish were euthanized by submersion in an ice water bath according to an NIH Animal Care and Use Committee-approved protocol, followed by full submersion into fresh 4% paraformaldehyde (PFA) and incubation for 24 h at 4 °C. Post-fixation, the fish were submerged in 0.5 M EDTA with a pH of 8.0 and incubated at room temperature for 7 days with gentle agitation. Before processing they were rinsed with 2 washes of nuclease-free PBS. The standard processing protocol for paraffin-embedded samples was used. Two fish were embedded in the same block, with one embedded sagittally and the other coronally. The coronal sections were captured using the bread loafing technique and placed in the block with the tail side down. The tail was removed just past the anus for the coronal sections only. Serial sections from the paraffin blocks were cut at 5 µm and placed on positively charged slides for H&E and multiplex fluorescent images using RNA in situ hybridization (RNAscope) and immunofluorescence. RNA-ISH RNAscope probes were acquired from Advanced Cell Diagnostics (Newark, CA). Histopathology was performed by a board-certified veterinary pathologist. Organs evaluated included the following: bone, brain, chromaffin tissue, corpuscle of stannous, esophagus, eye, gall bladder, gill, heart, kidney, hematopoietic tissue, intestine, liver, nares, oral cavity, ovary, pancreas, peripheral nerve, pseudobranch, skeletal muscle, skin, spinal cord, spleen, statoacoustic organ, and swim bladder. Tissue sections were deparaffinized twice in xylene for 5 min each and then twice in 100% ethanol for 3 min each. Tissue pretreatment consisted of antigen retrieval in 1X Dako Target Retrieval Solution (pH 9.0) in a RHS-1 Microwave Vacuum Histoprocessor (Milestone Medical, Kalamazoo, MI) at 10 min to 100 °C and 25 min at 100 °C. Sections were then manually stained with a RNAscope Multiplex Fluorescent v2 Kit (Advanced Cell Diagnostics) with either a 3-plex Negative control probe, 3-plex positive control probe or with a cocktail of Abcb5-C1 (Dr-Abcb5-C1, Cat# 493561) and Abcb4-C2 (Dr-Abcb4-C2, Cat# 493551-C2) probes with a 1:750 dilution of TSA-Fluorescein Plus and TSA-Cyanine 3 Plus (PerkinElmer, Shelton, CT), respectively. RNAscope staining was followed by the C219 IHC at a 1:200 dilution for 30 min on a Bond RX auto-stainer using the Bond Polymer Refine Kit (Leica, Buffalo Grove, IL) minus DAB & Hematoxylin. Antibody binding was detected with a 1:50 dilution of anti-HRP conjugated with Alexa 594 (Jackson ImmunoResearch Laboratories, Inc., West Grove, PA) for 30 min. Cells were counterstained with 4′,6-diamidino-2-phenylindole (DAPI). Selected organs were evaluated for semi-quantitative scoring for each marker. For colocalization studies with claudin-5, RNAscope staining was followed by claudin-5 (Invitrogen, Grand Rapids, NY, cat # 35-2500) IHC at a 1:50 dilution for 30 min on a Bond RX auto-stainer using the Bond Polymer Refine RED Kit (Leica) minus Hematoxylin.

## Supplementary Information

Supplementary Information.

## Data Availability

The datasets generated during and/or analyzed during the current study are available in the PubChem repository (please see the Results section for accession numbers).
