# Supplementary material for: Characterization and tissue localization of zebrafish homologs of the human ABCB1 multidrug transporter
Source: Sci Rep. 2021 Dec 17;11:24150. doi: 10.1038/s41598-021-03500-8 (PMC8683423; doi:10.1038/s41598-021-03500-8)
Supplement: Supplementary file 1 — Supplementary Information. [file 41598_2021_3500_MOESM1_ESM.pdf]

Supplementary Information

for

Characterization and tissue localization of zebrafish homologs of the human *ABCB1* multidrug transporter

Robert W. Robey,[1] Andrea N. Robinson,[1] Fatima Ali-Rahmani,[1] Lyn M. Huff,[1] Sabrina Lusvarghi,[1] Shahrooz Vahedi,[1] Jordan M. Hotz, [1] Andrew C. Warner,[2] Donna Butcher,[2] Jennifer Matta,[2] Elijah F. Edmondson, [2] Tobie D. Lee,[3] Jacob S. Roth,[3] Olivia W. Lee,[3] Min Shen, [3] Kandice Tanner,[1] Matthew D. Hall, [3] Suresh V. Ambudkar,[1] Michael M. Gottesman[1*]

[1]Laboratory of Cell Biology, Center for Cancer Research, National Cancer Institute, National Institutes of Health, Bethesda, MD

[2]Molecular Histopathology Laboratory, Frederick National Laboratory for Cancer Research, Frederick, MD

[3]National Center for Advancing Translational Sciences, National Institutes of Health, Rockville, MD

Table S1. Cross-resistance profile of known human P-gp substrates with zebrafish Abcb4 and Abcb5[a]

**Compound**	**GI_{50} Vector (µM)**	**GI_{50} MDR-19 (µM)**	**RR* MDR-19**	**GI_{50} Dr Abcb4 (µM)**	**RR* Dr Abcb4**	**GI_{50} Dr Abcb5 (µM)**	**RR* Dr Abcb5**
Bisantrene	0.016±0.0042	18.8±7.2	1175	1.9±0.56	119	0.37±0.04	23
Camptothecin	0.009±0.004	0.011±0.004	1	0.009±0.001	1	0.012±0.004	1
Doxorubicin	0.0092±0.0023	0.89±0.63	97	1.3±0.45	141	0.021±0.0072	2
Etoposide	0.25±0.062	10.0±0.25	40	2.9±0.62	12	4.6±1.8	18
Mitoxantrone	0.0053±0.0011	0.10±0.017	19	0.090±0.035	17	0.010±0.0036	2
Paclitaxel	0.0063±0.0012	1.7±0.83	270	2.5±0.75	397	0.79±1.05	125
Vinblastine	0.0029±0.0008	0.39±0.12	134	0.26±0.06	90	0.08±0.01	28

[a]All compounds were tested at least 3 times. Results presented are mean GI_{50} values +/- standard deviation.
*Relative resistance (RR) value is the ratio of the GI_{50} values of MDR-19, Dr Abcb4 or Dr Abcb5 cells to the GI_{50} value of empty vector (Vector) cells.

Table S2. Cross-resistance profile of selected compounds from high-throughput screening[a]

**Compound**	**GI_{50} Vector (μM)**	**GI_{50} MDR-19 (μM)**	**RR* MDR-19**	**GI_{50} Dr Abcb4 (μM)**	**RR* Dr Abcb4**	**GI_{50} Dr Abcb5 (μM)**	**RR* Dr Abcb5**
17-AAG	0.53±0.35	4.5±2.4	8	7.0±0.82	13	3.8±0.66	7
AT9283	1.9±0.26	14.0±5.3	7	19.3±1.2	10	20.7±8.3	11
KW-2478	0.44±0.31	88.3±53.5	200	140±26	318	37±27	84
Romidepsin	0.0026±0.0012	0.85±0.56	327	2.3±0.31	884	0.053±0.023	20
VX-680	3.0±0.71	14.8±7.5	5	36.5±2.9	12	8.1±1.8	3
YM-155	0.0027±0.00059	6.2±4.8	2296	375±66	138888	0.021±0.0059	8

[a]All compounds were tested at least 3 times. Results presented are mean GI_{50} values +/- standard deviation.
*Relative resistance (RR) value is the ratio of the GI_{50} values of MDR-19, Dr Abcb4 or Dr Abcb5 cells to the GI_{50} value of empty vector (Vector) cells.

Table S3. Expression pattern of zebrafish *abcb4* and *abcb5*[a]

**Organ**	***abcb4***	***abcb5***
Brain	+++	-
Liver	+++	+
Kidney*	++	++
Skin	-	+++
Ovary	+	+++
Intestine	+++	-
Gill	-	+++

[a]Selected organs were evaluated for semi-quantitative scoring for each marker.
*Distinct regions of the nephron are positive for either *abcb4* or *abcb5* (not both).

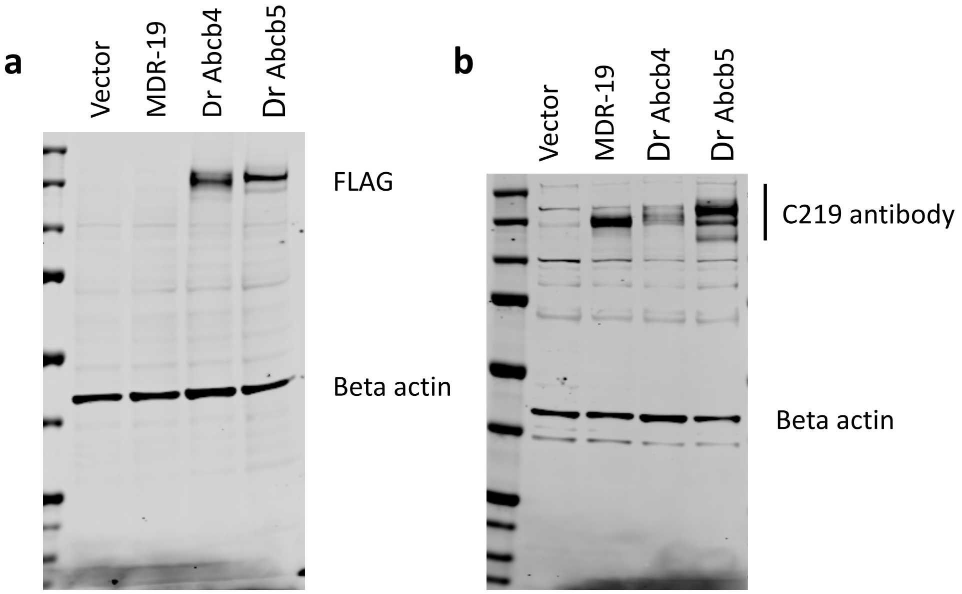

Figure S1. Full blots used in Fig. 1. Whole cell lysates were prepared and separated via PAGE as outlined in the legend to Fig. 1. Blots were probed with anti-FLAG antibody and beta actin (a) or anti-ABCB1 antibody C219 and beta-actin (b).

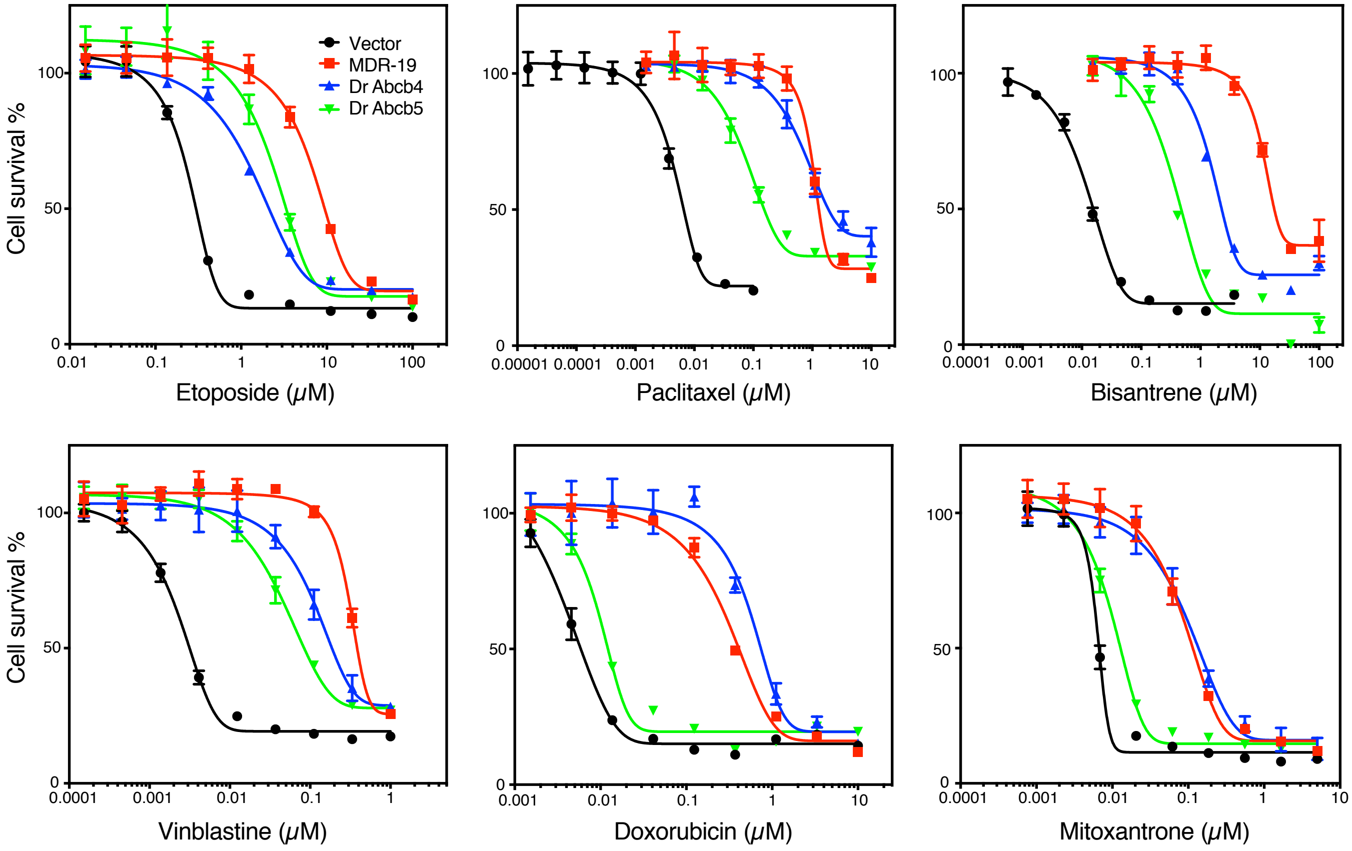

Figure S2. Zebrafish Abcb4 and Abcb5 confer differential resistance to known P-gp substrates. Three-day cytotoxicity assays were performed with the known human P-gp substrates etoposide, paclitaxel, bisantrene, vinblastine, doxorubicin and mitoxantrone on HEK-293 cells transfected with empty-vector cells (Vector, black curve) or cells expressing human P-gp (MDR-19, red curve), zebrafish Abcb4 (Dr Abcb4, blue curve), or zebrafish Abcb5 (Dr Abcb5, green curve). GI50 values were obtained from the curves and are summarized in Table S1.

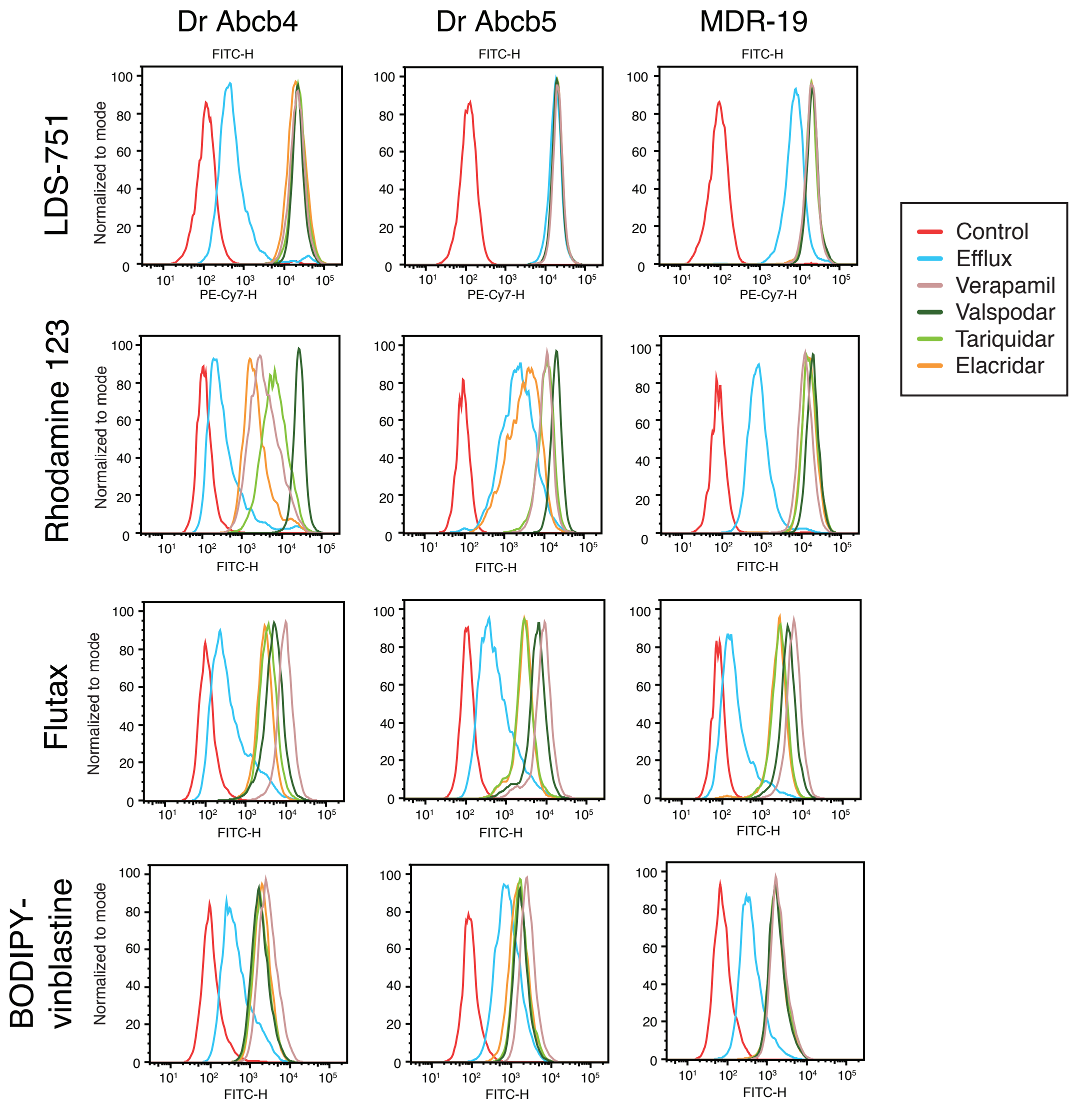

Figure S3. Zebrafish Abcb4 and Abcb5 differentially transport fluorescent P-gp substrates. HEK293 cells transfected to express zebrafish Abcb4 (Dr Abcb4), zebrafish Abcb5 (Dr Abcb5) or human P-gp (MDR-19) were incubated in medium with 0.5 μM LDS 751, 0.5 μg/ml rhodamine 123, 5 μM Flutax or 250 nM BODIPY vinblastine in the presence or absence of 10 μM elacridar, 10 μM tariquidar, 10 μM valspodar, or 100 μM verapamil for 30 min. The medium was removed and replaced with substrate-free medium in the presence or absence of inhibitor for an additional 1 h.

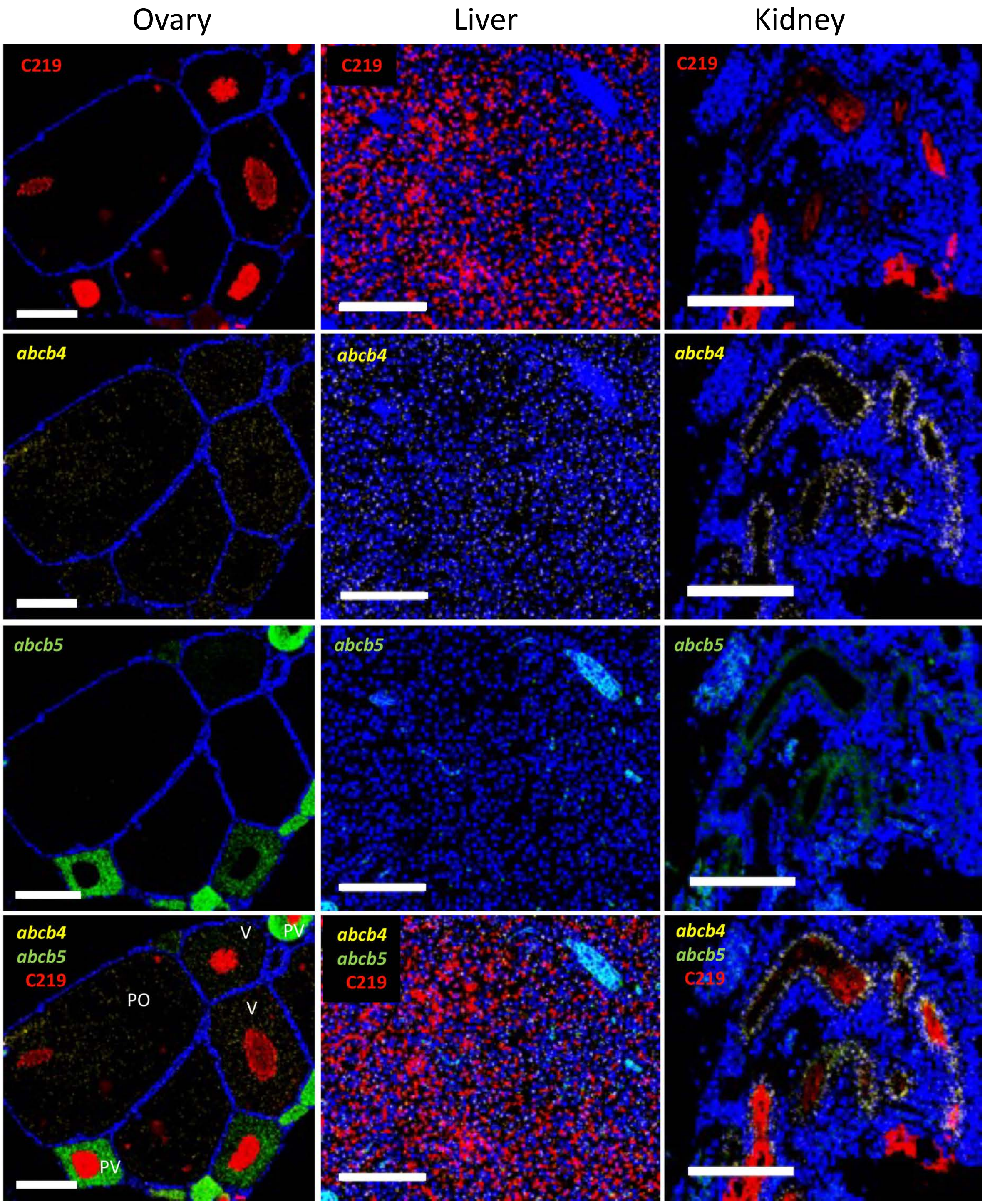

Figure S4. Zebrafish *abcb4* and *abcb5* RNA-ISH with C219 immunolabeling in the ovary, liver, and kidney of adult zebrafish. Zebrafish sections were stained with RNAscope® abcb4 (yellow) and *abcb5* (green) probes followed by the C219 antibody (red) as outlined in Materials and Methods. In the ovary, unique staining patterns are observed for different follicular stages. While *abcb4* is expressed throughout all follicular stages, *abcb5* is expressed at high levels in early pre-vitellogenic (PV) stages and lost at later stage (V, vitteloginic; PO, preovulatory follicles). Liver and kidney express predominantly abcb4. Fluorescence channels were interrogated individually and merged. Bars = 100 µm. Nuclei were stained with DAPI (blue).

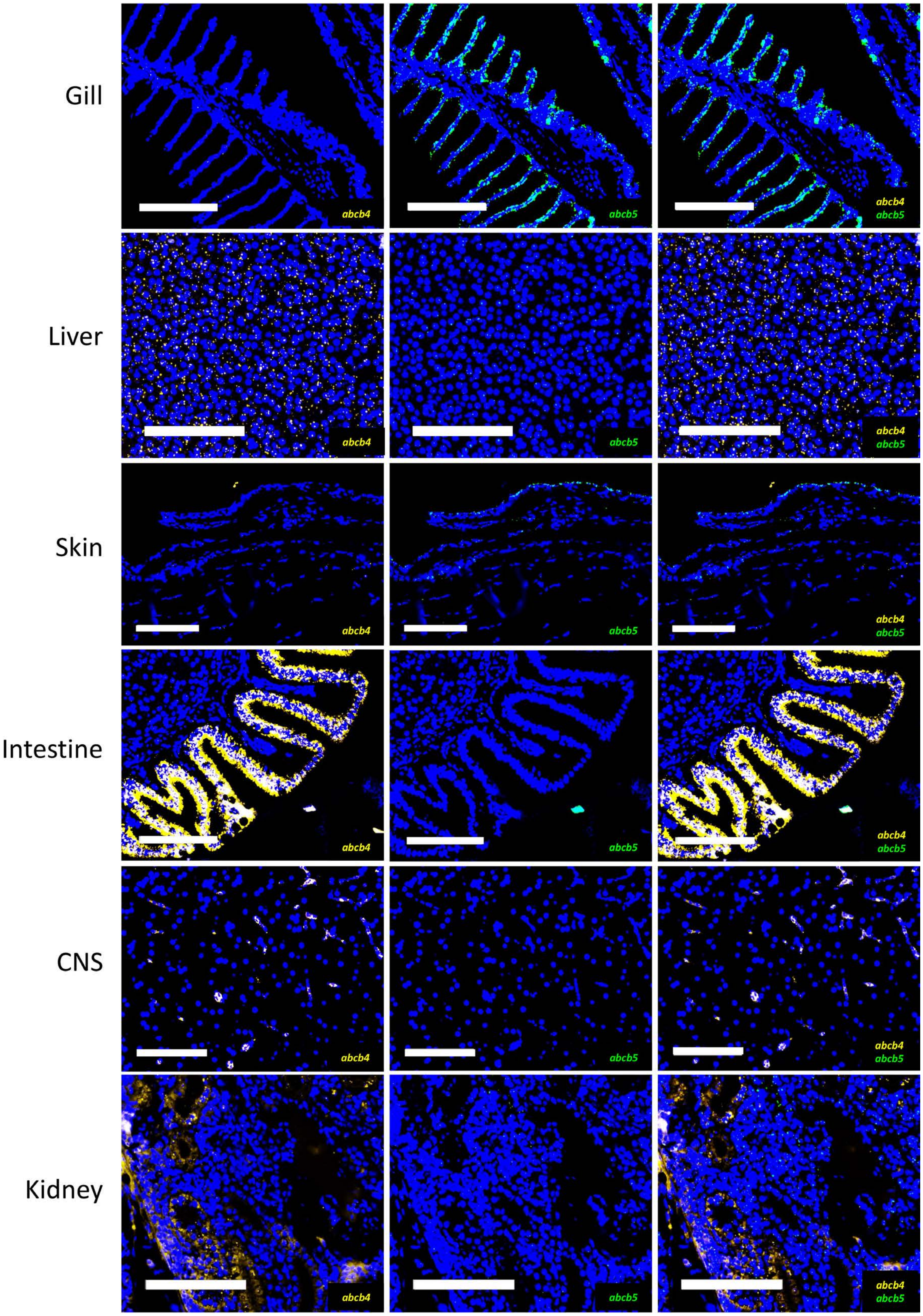

Figure S5. Zebrafish abcb4 and abcb5 signal following RNA-ISH. Multiplex RNA-ISH for abcb4 (yellow) and abcb5 (green) is performed as outlined in Materials and Methods. Nuclei were stained with DAPI (blue). Fluorescence channels were interrogated individually and merged. Bars = 100 μm.
